# The Impact of Sex Hormones on Keratoconus

**DOI:** 10.3390/jcm14124365

**Published:** 2025-06-19

**Authors:** Konstantinos Droutsas, Iasonas Makrypoulias, Irini Chatziralli, Konstantinos Panagiotopoulos, Maria-Evanthia Sotirianakou, Dimitris Papaconstantinou

**Affiliations:** 1 1st Department of Ophthalmology, Geniko Nosokomeio Athenon Giorgos Gennematas, National and Kapodistrian University of Athens, 11527 Athens, Greece; kondrou@med.uoa.gr (K.D.); anthistk@med.uoa.gr (M.-E.S.); dimpapac@med.uoa.gr (D.P.); 2 2nd Department of Ophthalmology, Panepistemiako Geniko Nosokomeio Attikon, National and Kapodistrian University of Athens, 12462 Athens, Greece; eirchat@med.uoa.gr; 3 Biochemistry Laboratory, General Hospital of Athens ‘Georgios Gennimatas’, 11527 Athens, Greece; biochemical@gna-gennimatas.gr

**Keywords:** keratoconus, sex hormones, estrogen, androgen, prolactin, extracellular matrix remodeling, corneal ectasia

## Abstract

Keratoconus (KC) is a progressive non-inflammatory disorder characterized by significant changes in the corneal structure, leading to severe vision loss. Risk factors include eye rubbing, a positive family history, and allergic reactions. There is growing evidence suggesting that sex hormones may influence the development and progression of KC, but the exact mechanisms and extent of their impact remain unclear and controversial. This review aims to examine the current literature on the association between KC and sex hormones and to evaluate the potential of these hormones as clinical markers for diagnosing, prognosticating, and managing KC.

## 1. Introduction

Keratoconus (KC) is a non-inflammatory progressive disorder characterized by alterations in corneal morphology, leading to visual impairment and potentially progressing to severe visual loss. It is classified within the group of ectatic corneal diseases (ECDs), which are marked by the progressive thinning and subsequent bulging of the corneal structure. Other phenotypes within the ECD spectrum include pellucid marginal degeneration (PMD), keratoglobus, and post-refractive surgery progressive corneal ectasia [1,2]. A recent meta-analysis estimated the prevalence of KC in the general population to be 1.38 per 1000 individuals, with a slightly higher prevalence in men (20.6 per 1000) compared to women (18.33 per 1000) in studies that reported sex. The same study identified eye rubbing, a positive family history, allergies, asthma, and eczema as primary risk factors for the development of KC [3].

Sex hormones are well studied as essential regulators of multiple physiological processes, primarily affecting reproductive functions but also exerting significant influence on other tissues. The eye exhibits sensitivity to sex hormones, with emerging evidence suggesting that these molecules play a crucial role in maintaining the normal structure and function of the cornea [4,5,6]. In addition, many studies have reported structural and functional alterations of the cornea occurring under significant hormonal events such as pregnancy [4,7,8,9,10,11] and menopause [4,12]. Also, the use of external hormones used for birth control, hormonal stimulation prior to in vitro fertilization, and hormone replacement therapy has also be linked to corneal changes [4,13,14,15,16,17].

This review aims to explore the potential role of sex hormones in the development and progression of KC, as well as their potential use as seromarkers for the diagnosis and management of this disease.

## 2. Materials and Methods

A comprehensive literature search was conducted using PubMed to identify peer-reviewed articles examining the role of sex hormones in the pathogenesis and progression of keratoconus. The search strategy employed a combination of keywords and medical subject headings (MeSH), including (“keratoconus”, “corneal ectasia”, “corneal ectatic disorders”) AND (“hormones”, “LH”, “FSH”, “DHEAS”, “testosterone”, “prolactin”, “progesterone”, “estrogens”, “E1”, “E2”, “E3”). The search was limited to human studies published in English and excluded animal research and case reports. The search included all the relevant literature published up to June 2024.

### 2.1. Study Selection and Data Extraction

Titles and abstracts were screened to assess relevance, and full-text articles were reviewed when eligibility was unclear. Studies were included if they reported original data regarding hormone levels in patients with keratoconus or investigated associations between hormonal biomarkers and keratoconus-related clinical features. Extracted data included sample size, patient demographics, hormone types analyzed, as well as key findings on hormone–keratoconus relationships.

The selection process followed a structured screening strategy, and discrepancies were resolved through discussion among reviewers. Data were synthesized narratively considering the heterogeneity in study designs and hormone assessment methods.

### 2.2. Data Synthesis

The included studies varied in terms of hormone types, sample sources (e.g., plasma, saliva, tears, etc.), and corneal parameters evaluated. The synthesis highlights recurring patterns, conflicting findings, and potential mechanisms linking sex hormones with keratoconus pathophysiology, with the goal of outlining current evidence and identifying areas for future research.

## 3. Hormonal Involvement in Keratoconus

### 3.1. Estrogens

Estrogens play a critical role in cellular proliferation, differentiation, and apoptosis. Different studies have demonstrated the expression of estrogen receptors in the human cornea [6,18,19,20,21,22]; however, the exact role of estrogens in the pathogenesis of KC remains controversial.

Zhao et al. compared the plasma levels of various sex hormones, including estriol and estradiol, between 62 patients with KC and 120 patients with mild-to-moderate myopia and astigmatism scheduled for laser vision correction (LVC). Males with KC demonstrated higher levels of estradiol (E2) compared to those in the LVC group (143.75 ± 34.82 pmol/L vs. 124.80 ± 43.56 pmol/L; *p* = 0.013). In addition, a weak positive correlation was observed between E2 levels and maximum simulated keratometry (Kmax) (r = 0.222, *p* = 0.007), which was used as a corneal morphological parameter [23].

In a different study, hormone levels were measured in the saliva of 64 KC patients and compared to those of 14 healthy controls. The findings revealed a significant reduction in estrone (E1) levels (1.3-fold, *p* = 0.0222) in KC patients, while only a slight decrease in estriol (E3) and 17β-estradiol levels was observed. The study also investigated the relationship between KC severity and altered hormone levels. E1, E3, 17β-estradiol, and DHEA-S were analyzed in correlation with age, KC grade, Kmax, and minimum central corneal thickness (CCtmin). Notably, 17β-estradiol levels demonstrated higher variability with age and were slightly lower in those with more severe KC (grades > 2), high Kmax (>50), and CCTmin (<425 μm) [24].

In a prospective observational study involving 28 adults with KC scheduled for cornea cross-linking (CXL) treatment, the researchers assessed the clinical characteristics of the treated eye compared to the untreated eye (control). Based on the hypothesis that baseline hormone levels could influence the effectiveness of the treatment in terms of corneal curvature and central corneal thickness, plasma levels of DHEA-S, E1, and E3 were measured before and after CXL. Estrogen and estriol levels decreased in the majority of patients (52% and 56%, respectively), while only 22% of patients showed a reduction in DHEA-S levels. Regarding potential associations between circulating hormone levels and corneal parameters, E3 levels exhibited a significant positive correlation with the highest baseline Kmax (Spearman’s r = 0.55, *p* = 0.01) but no significant correlation with CCtmin [25].

Sharif R and his team compared plasma, saliva, and tear samples of 147 KC patients with 60 healthy controls. Their results also showed a significant downregulation in both E1 (*p* < 0.0001) and E3 (*p* < 0.0001) levels in KC plasma and saliva samples. On the other hand, no significant differences were observed in 17β-estradiol levels [26]. Their results further demonstrate the potential link between KC and systemic estrogen levels.

### 3.2. Androgens

The role of androgens in KC has been widely investigated, with scientific evidence demonstrating the expression of androgen receptors (ARs) in the human cornea, implementing their potential role in corneal physiology [6,21,27]. Different studies indicated higher expression levels of AR in patients with KC [19,20]. A cross-sectional study compared the serum levels of sex hormones between 76 patients with KC and 26 normal control individuals. The results revealed statistically significant elevated levels of dehydroepiandrosterone sulfate (DHEAS) (3.71 ± 2.23 vs. 2.53 ± 1.77 µg/mL; *p* = 0.009) and testosterone (T) (6.18 ± 3.80 vs. 1.57 ± 1.76 ng/mL; *p* < 0.001) in male KC patients compared to controls. In female patients, although both DHEAS and T levels were higher, only the T levels reached statistical significance (0.78 ± 0.96 vs. 0.32 ± 0.13 ng/mL; *p* < 0.001) [28]. The higher concentration of DHEAS in the saliva and plasma of patients with KC, compared to individuals without KC, has also been demonstrated in other studies [24,26]. Although the aforementioned studies indicate a positive correlation between the occurrence of KC and androgen levels, other studies present conflicting findings. Zhao et al. found that T levels were significantly lower in both male and female patients with KC when compared to the levels of the LVC group. Additionally, female patients showed a positive correlation between T levels, the central corneal thickness, and the thinnest corneal thickness [23]. On the other hand, Van L. et al. found no significant correlation between DHEAS levels and the highest Kmax or lowest CCtmin in their KC patients [25]. Collectively, these conflicting findings, that may be attributed to variations in sample collection procedures and differences in the ethnic composition of the studied populations, as well as the limited number of KC subjects included in the studies and the bioavailability of sex hormones, highlight a complex relationship between androgens and KC. This suggests that further research is needed to fully understand the role of these hormones in the pathophysiology of the condition.

### 3.3. Prolactin

Prolactin (PRL) is primarily associated with lactation and has been identified as a potential player in KC pathogenesis. Jamali et al. compared the serum levels of PRL between 76 patients with KC and 26 normal control individuals. They reported elevated mean serum PRL levels (13.33 ± 17.85 vs. 4.63 ± 3.10 ng/mL; *p* < 0.001) in women with KC compared to controls [28]. A different study measured and compared the concentration of PRL in the aqueous humor of 100 KC patients who underwent penetrating keratoplasty and 100 patients who were surgically treated for cataracts. The results showed lowered levels of prolactin in the aqueous humor of patients with KC (3.18 ± 0.34 ng/mL vs. 3.33 ± 0.32 ng/mL in cataract patients) [29]. These findings indicate that prolactin may have a more complex role in corneal health, potentially driving pathological changes in KC.

### 3.4. Gonadotropins: LH and FSH

Emerging research has identified gonadotropins as potential regulators in keratoconus. It has been proposed that these hormones, typically involved in reproductive health, may influence corneal cells and the extracellular matrix (ECM), as the human cornea expresses receptors for both of them [30,31]. Karamichos’s group, from the University of Oklahoma Health Sciences Center, measured the level of LH and FSH in the plasma of 86 patients with KC (63 males, 23 female) and 45 healthy controls (22 male, 23 female). Their results showed no significant difference in the levels of LH and FSH between the two groups; however, they marked a difference in the LH/FSH ratio, which appeared significantly lower, especially in males of the KC group when compared to healthy controls [30]. Similarly, Jamali’s team also reported non-significant alterations in the plasma levels of these hormones between KC patients and the control group [28]. Nevertheless, more research is required to find out if they can be used as a target for potential treatments or provide a tool for early diagnosis or/and prognosis for keratoconus.

### 3.5. Progesterone

Progesterone has also been a subject of research regarding their role in eye diseases. Progesterone receptors exist in many parts of the eye, including the cornea [19,21,22]. Zhao et al. reported no significant difference in the level of progesterone in the plasma of a group of 62 patients with KC compared to an LVC group [23]. Despite these findings, and as Zhao X’ team also suggests, we believe that more research should be performed in order to decisively conclude if progesterone plays an active role to keratoconus appearance and/or progression.

Table 1 contains a summary of the aforementioned findings regarding the levels of the various sex hormones.

## 4. Molecular Mechanisms

The molecular mechanisms involved in the interaction between sex hormones and KC remains unclear. One possible way they promote the progression of KC is by influencing the expression of matrix metalloproteinases (MMPs). MMPs are a group of 24 zinc-dependent enzymes capable of breaking down collagen and other ECM proteins, essential structural elements of the cornea. MMP-driven extracellular ECM remodeling is considered a crucial step in corneal healing. However, maintaining a precise balance in MMP expression is mandatory to preserve the cornea’s integrity and transparency, as well as ensuring proper healing. An imbalance in this carefully regulated process could, over time, lead to progressive weakening of the cornea [32,33,34]. Philipp Anders et al. suggested that high levels of prolactin reduced the IL-8 and IL-6 secretion in KC corneal stromal fibroblasts (CSFs) compared to normal CSFs [35], providing a potential link between the role of prolactin and the role of cytokines in the context of KC. Hongbo Yin et al. reported that treatment with 17β-estradiol significantly decreased both the mRNA expression and protein levels of matrix metalloproteinase-2 in human corneal stromal cells [36]. In contrast, Suzuki et al. suggested that 17β-estradiol enhances the expression of proinflammatory cytokines and MMP genes in human corneal epithelial cells, significantly upregulating IL-1β, IL-6, IL-8, GM-CSF, and MMP-2, -7, and -9 mRNAs compared to a placebo [37].

The role of prolactin-induced protein (PIP) was also examined, and it was proposed as a novel biomarker for KC [26]. There is evidence showing lower levels of PIP expression in the tears, saliva, and plasma of patients with KC when compared to healthy controls [26,38], providing their use as a potential tool for early diagnosis and monitoring disease progression. PIP has also been shown to be positively impacted by CXL performed on stromal cells isolated from keratoconus donors [39].

Escandon et al.’s team used an established 3D in vitro model to show that healthy cornea stromal cells (HCFs) and KC cornea stromal cells (HKCs) responded differently when stimulated with various concentrations of E1 and E3. The results suggested that E1 and E3 play a role in the regulation of ER, AR, and the progesterone receptor (PR) [18]. Collectively, these findings add to a growing amount of evidence concluding that the regulation of sex hormones and their receptors seem to play a crucial role in KC development, suggesting that prolonged hormonal abnormalities from birth may drive the manifestation of the disease.

## 5. Limitations

The reviewed literature presents several limitations. Although research over the past two decades has increasingly explored the connection between keratoconus (KC) and sex hormones, many studies involve small sample sizes, which limits the ability to generalize findings across broader populations. Variability among study populations—such as differences in genetic backgrounds, environmental exposures, and sex hormone bioavailability—further complicates the interpretation of results, making it challenging to isolate the specific impact of sex hormones on KC. Additionally, the lack of longitudinal studies hinders the assessment of long-term hormonal fluctuations on KC progression.

## 6. Conclusions

The body of research reviewed highlights the multifaceted impact of sex hormones on keratoconus. Understanding these hormonal influences may offer valuable insights into potential diagnostic markers and therapeutic strategies. Due to limitations of the current data, future research should continue to explore these relationships, aiming to translate these findings into clinical practice to improve outcomes for keratoconus patients.

## Figures and Tables

**Table 1:** Summary of levels of various sex hormones in subjects with keratoconus versus non-keratoconus subjects.

	No. KC Sub	LH	FSH	LH/FSH Ratio	E1	E2	Ε3	Prolactin	DHEAS	Testosterone	Progesterone
[23]	62					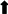 Men plasma				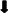 Men and women plasma	ns
[24]	64				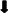 In saliva	Ns in saliva			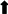 In saliva		
[25]	28				Ns correlation with the highest Kmax; lowest CCTmin		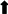 Plasma with the highest Kmax		Ns correlation with the highest Kmax; lowest CCtmin		
[26]	147				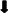 In plasma and saliva	Ns in plasma and saliva	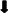 In plasma and saliva		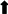 In plasma and saliva		
[28]	76	ns	ns					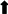 Women plasma	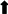 Men plasma	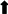 Men and women plasma	
[29]	100							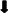 In aqueous humor			
[30]	86	ns	ns	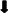 In plasma							

Ns: non-significant, E1: estrone, E2: estradiol, E3: estriol, LH: luteinizing hormone, FSH: follicle stimulation hormone, DHEAS: dehydroepiandrosterone sulfate, No. KC Sub: number of subjects with keratoconus, Kmax: maximum simulated keratometry, CCTmin: minimum central corneal thickness, 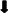: downregulated, 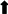: upregulated.

## Abbreviations

The following abbreviations are used in this manuscript:

KC	Keratoconus
ECD	Ectatic Corneal Diseases
PMD	Pellucid Marginal Degeneration
LVC	Laser Vision Correction
CXL	Corneal Cross-Linking
Kmax	Maximum Simulated Keratometry
CCtmin	Minimum Central Corneal Thickness
AR	Androgen Receptor
PRL	Prolactin
LH	Luteinizing Hormone
FSH	Follicle-Stimulating Hormone
DHEAS	Dehydroepiandrosterone Sulfate
E1	Estrone
E2	17β-Estradiol
E3	Estriol
T	Testosterone
PR	Progesterone Receptor
PIP	Prolactin-Induced Protein
MMPs	Matrix Metalloproteinases
ECM	Extracellular Matrix
CSFs	Corneal Stromal Fibroblasts
HCFs	Healthy Corneal Stromal Fibroblasts
HKCs	Keratoconus Corneal Stromal Cells
IL-6	Interleukin 6
IL-8	Interleukin 8
